# Mechanisms and Pathogenesis of Diabetic Cognitive Impairment: A Comprehensive Review

**DOI:** 10.14336/AD.2025.0769

**Published:** 2026-07-22

**Authors:** Chang Qi, Ming Qin, Yu Wang, Cong Li, Huiying Yan, Lina Feng, Mingquan Li

**Affiliations:** ^1^College of Traditional Chinese Medicine, Changchun University of Chinese Medicine, Changchun, China.; ^2^Department of Neurology, the Third Affiliated Clinical Hospital of the Changchun University of Chinese Medicine, Changchun, China.; ^3^Department of Neurology, the Second Affiliated Hospital of Shandong First Medical University, Shandong First Medical University & Shandong Academy of Medical Sciences, Taian, China.

**Keywords:** Diabetic Cognitive Impairment, DCI, Cognitive Impairment, Diabetes Mellitus, Mechanism

## Abstract

Diabetic cognitive impairment (DCI) is a form of cognitive dysfunction that affects individuals with diabetes, marking it as one of the complications linked to this disease. This condition typically presents as deficits in various cognitive abilities, such as memory, learning, language, motor skills, perception, and attention. Studies show that around 13% of diabetic individuals aged 65 to 74 experience cognitive impairment, with this figure rising to 24% for those over 75. As the global incidence of DCI increases, the economic and caregiving challenges for both individuals and society are also growing. The specific mechanisms underlying DCI remain unclear, and the relationships among various pathological processes are still under investigation. The study of DCI mechanisms continues to present numerous unresolved mysteries, such as unclear causal relationships: does metabolic disorder (e.g., hyperglycemia) directly damage neurons, or does it indirectly affect cognition through vascular lesions? Additionally, the mechanisms of individual heterogeneity pose further questions: why do some diabetic patients experience cognitive decline (CD) while others do not? Therefore, understanding the pathological alterations and the fundamental reason behind DCI is essential for improving early prevention and treatment strategies for individuals exhibiting clinical symptoms of this disorder. Furthermore, DCI represents a significant intersection between metabolic and neurodegenerative diseases, which encourages the integration of cognitive assessments into routine diabetes management. This article not only provides a systematic review of existing research but also serves as a bridge connecting basic science with clinical practice, offering theoretical support for the precise prevention and early diagnosis of DCI in patients.

## 1. Introduction

Diabetes mellitus (DM) is a long-term metabolic condition primarily characterized by elevated blood glucose levels. This can significantly complicate the lives of many individuals. Even with appropriate medical care, those affected may experience both immediate and long-term health challenges, which can adversely affect their quality of life. The diverse complications associated with DM, coupled with the necessity for continuous medical attention and lifestyle modifications, render DM a critical health concern. Additionally, the financial burden of this condition extends beyond the patients themselves, impacting on their families and the wider community. The global incidence of diabetes is on the rise, with projections suggesting it could reach 642 million individuals by 2040 [1]. In 2021, the worldwide expenses related to diabetes management were estimated at $966 billion, with predictions indicating an increase to $1,054 billion by 2045 [2]. Studies indicate that CD affects as many as 13% of diabetics aged 65 to 74, with this percentage rising to 24% among those aged 75 and older [3].

Diabetic cognitive impairment (DCI) represents a major complication of DM. This condition primarily results from extended periods of high blood glucose level, which damages small blood vessels and leads to both short-term and long-term cognitive difficulties. DCI is characterized by issues with language abilities, visual memory, processing speed, and executive functions related to diabetes. In 2021, the American Diabetes Association (ADA) formally acknowledged DCI as a prevalent complication of Type 2 Diabetes Mellitus (T2DM), the most common form of diabetes. ADA guidelines also suggest that cognitive impairment (CI) may progressively worsen. Additionally, inadequate glycemic control has been associated with cognitive deterioration, and the length of time a person has diabetes is inversely related to cognitive function [4]. Research indicates that individuals with T2DM face a significantly higher risk of CI compared to the general population. Specifically, the likelihood of developing Alzheimer's disease (AD), vascular dementia (VaD), and mild cognitive impairment (MCI) is 1.46, 1.48, and 1.21 times greater, respectively, for those with diabetes compared to non-diabetics. Furthermore, an earlier diagnosis of diabetes correlates with a heightened risk of dementia in these patients [5, 6].

**Figure 1.**
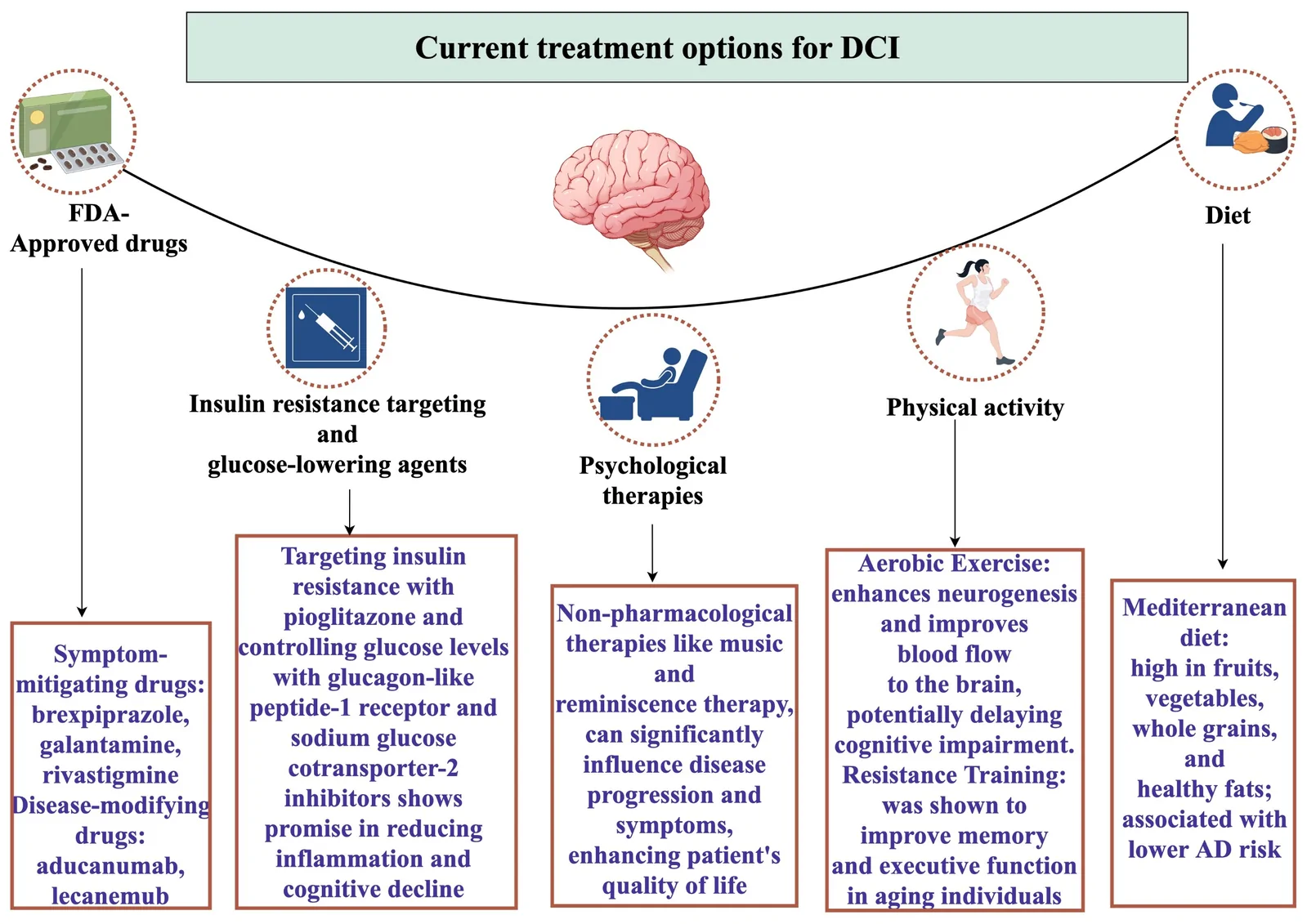
**Current treatment options for DCI**. GLP-1 receptor agonists, including liraglutide, semaglutide, and exenatide, exert neuroprotective effects by enhancing insulin sensitivity, reducing neuroinflammation, and promoting neuronal survival. SGLT-2 inhibitors, such as empagliflozin and dapagliflozin, function by decreasing glucose reabsorption in the kidneys, thereby improving glycemic control in patients with T2DM and demonstrating potential benefits in the treatment of AD. Additionally, psychological therapies, including music therapy and reminiscence therapy, have been shown to alleviate symptoms and improve the quality of life for patients.

With the growing number of older adults and the increasing incidence of DM, the forecasted figures for individuals likely to suffer from DCI are on the rise. As DM and CI become more widespread globally, concerns about DCI are intensifying. Fluctuations in glucose levels among diabetic patients can lead to significant alterations in brain structure, such as reduced gray matter volume, neuronal injury, cortical shrinkage, and an overall decrease in brain size [7]. Those with diabetes show a notable decline in gray matter in areas like the prefrontal cortex, parahippocampal gyrus, cerebellar cortex, brainstem, and tonsillar regions of the cerebellum when compared to healthy individuals [8]. Findings from a cross-sectional study suggest that diminished gray matter volume may play a role in the connection between DM and cognitive abilities [7]. Furthermore, white matter degeneration in diabetic patients is primarily noted in the frontal and temporal lobes, correlating with deficits in visuospatial working memory, planning, and processing speed [8]. A meta-analysis indicates that the worldwide prevalence of DCI could reach up to 45% [9]. Once individuals with DCI progress to dementia, their cognitive abilities become permanently compromised. Clinically, the limited insight into the root causes of DCI has resulted in a scarcity of targeted pharmacological interventions. Current treatment approaches largely rely on hypoglycemic agents (e.g., metformin, thiazo-lidinediones, sulfonylureas) and drugs designed to improve cognitive function (e.g., cholinesterase inhibitors, memantine) [10] (Fig. 1). However, these treatments frequently cause gastrointestinal issues and extrapyramidal side effects, highlighting the need for enhancements in both their effectiveness and safety. Therefore, there is a pressing need for more comprehensive research into the mechanisms behind DCI. This study aims to explore the pathological changes and processes associated with DCI, laying the groundwork for future investigations and clinical applications.

## 2. Current Status of DCI Treatment

Current treatments for DCI primarily focus on lifestyle interventions, glycemic control, and pharmacological therapies targeting dementia and AD. Dietary control and exercise may enhance the neuronal plasticity in brain regions associated with cognitive function in patients with DCI [11]. This includes advocating for a Mediterranean diet (such as olive oil, vegetables, fruits, fish, seafood, and legumes) and maintaining a healthy circadian rhythm as part of lifestyle modifications [12-19]. Antidiabetic medications can improve hyperglycemia, insulin resistance (IR), and cellular metabolism, and they can combat tissue inflammation and oxidative stress (OS) associated with IR states. They may also positively influence cellular metabolism in the brain and enhance cognitive abilities. Research indicates Metformin and GLP-1 receptor agonists (GLP-1RAs) have the potential to enhance cognitive function and reduce the risk of dementia. Sulfonylureas, thiazolidinediones, dipeptidyl-peptidase IV inhibitors (DPP-4i), and sodium-dependent glucose transporters 2 inhibitors (SGLT2i) may have protective effects on cognitive function, although clinical research evidence is still insufficient, and further studies are needed to confirm these findings. A controlled study indicated that long-term use of Sulfonylureas does not alter the overall risk of developing AD [20]. There are also reports suggesting a slight increase in the incidence of dementia among patients who have been on long-term metformin therapy [21]. Additionally, some studies have confirmed that after a 40-month treatment period, the Thiazolidinedione group exhibited significant CI compared to other treatment groups [22]. The relationship between DPP-4 inhibitors and cognitive dysfunction in diabetes is currently supported only by animal studies, with no relevant clinical research available. Recent studies have shown that Empagliflozin significantly improves CD in T2DM mice by controlling blood glucose; however, related evidence remains limited [23]. Currently, there are no clinical trials using this medication for the treatment of AD.

Currently, there are no relevant clinical trials on the treatment of DCI. Existing treatments primarily target general AD, and no therapy has yet been proven effective in alleviating disease progression. The current therapeutic approaches are mainly symptomatic treatments and disease-modifying therapies(DMT), which offer limited clinical benefits [24]. Symptomatic treatment drugs aim to improve cognitive function and control neuropsychiatric symptoms in patients, compensating for neuronal damage by enhancing the activity of specific neurotransmitters in the brain. The symptomatic treatment drugs approved by the U.S. Food and Drug Administration (FDA) for AD primarily include cholin-esterase inhibitors (such as donepezil, rivastigmine, and galantamine) and N-methyl-D-aspartate (NMDA) receptor antagonists (such as memantine). However, these drugs cannot prevent or reverse disease progression. DMTs aim to delay disease progression by intervening in the pathophysiological processes of AD, such as the abnormal changes in Aβ or tau proteins [25]. Although some progress has been made in recent years, the overall results remain unsatisfactory. While most new drugs have shown certain efficacy in early clinical trials, their long-term clinical benefits have not been confirmed in large-scale clinical trials. Furthermore, the high costs and serious adverse reactions also limit the widespread use of these drugs. Therefore, we must continue our efforts to seek new breakthroughs and provide more and better treatment options for patients with DCI.

## 3. Pathogenesis of DCI

### 3.1 Glycaemic variability (GV)

GV is a critical component of glucose homeostasis. Although it has not yet been definitively established as an independent risk factor for diabetes complications, GV may indicate the presence of excessive glycaemic excursions and, consequently, the risk of hyperglycaemia or hypoglycaemia. Currently, GV is defined by a growing number of metrics, representing either short-term (within-day and between-day variability) or long-term GV, typically based on serial measurements of HbA1c or other glycaemic indicators over an extended period. Yu et al. [26]conducted a pooled analysis of two prospective population-based cohorts: the Health Retirement Study (HRS) and the English Longitudinal Study of Ageing (ELSA). They demonstrated that, in the overall sample, participants in the highest quartile of HbA1c variability exhibited a significantly greater rate of memory decline (-0.094 SD/year, 95% CI -0.185, -0.003) and executive function decline (-0.083 SD/year, 95% CI -0.125, -0.041) compared to those in the lowest quartile, regardless of mean HbA1c values over time. Zheng et al. [27]included 5,189 participants with baseline HbA1c levels ranging from 15.9 to 126.3 mmol/mol. They revealed that a 1 mmol/mol increase in HbA1c was significantly associated with an accelerated decline in global cognitive z-scores (-0.0009 SD/year, 95% CI -0.0014, -0.0003), memory z-scores (-0.0005 SD/year, 95% CI -0.0009, -0.0001), and executive function z-scores (-0.0008 SD/year, 95% CI -0.0013, -0.0004) after adjusting for diabetes risk factors.

Previous studies have shown that GV is closely associated with atherosclerosis [28]. Research by TANG et al. [29] has confirmed that patients with T2DM and high GV experience more severe CD. Firstly, elevated GV can lead to increased adhesion of inflammatory factors and macrophages to vascular endothelial cells, exacerbating endothelial dysfunction [30]. Concurrently, individuals with high GV may also suffer from other pathogenic factors such as hypertension and IR, which further elevate the risk of cerebrovascular events. Once the structure of the cerebrovascular system is compromised, it leads to dysregulation of cerebrovascular function, resulting in the occurrence of amyloid angiopathy, which continuously diminishes cognitive function in patients, ultimately leading to CI [31]. Additionally, other studies have found that the mechanism by which GV affects cognitive function may involve pathological changes damaging brain cells in cognitive areas such as the hippocampus, cerebellum, and frontal lobe, resulting in decreased cognitive abilities. Subcortical infarcts caused by GV disrupt the network connections between the basal ganglia and the dorsolateral prefrontal cortex, severing the fiber connections between certain subcortical structures and the prefrontal cortex, frontal cortex, and cingulate gyrus, thereby inhibiting some cognitive-related functions in the frontal cortex [32].

### 3.2 IR and Insulin deficiency

Insulin, a hormone composed of proteins primarily produced by the pancreatic β-cells, serves an essential function as a neurotrophic agent [33]. Research has shown that insulin can penetrate the blood-brain barrier (BBB) [34] and is present in several brain regions, including the cortex, hippocampus, and hypothalamus [35]. Various neuronal cell types in the human brain have insulin receptors, and different insulin variants can elicit unique responses in distinct brain areas [36].The impact of insulin on CD related to diabetes has garnered significant interest. DM is characterized by IR and deficiency, with the former being prevalent in T2DM and the latter mainly occurring in T1DM. In T2DM, the body's initial response to IR often leads to increased insulin levels in the blood. The Homeostasis Model Assessment of Insulin Resistance (HOMA-IR) is a tool used to assess IR in patients. Research has revealed a significant correlation between HOMA-IR scores and glycated hemoglobin (HbA1c) levels concerning cognitive deterioration in diabetic individuals [37]. Additionally, another study found that higher plasma insulin levels and IR were linked to reduced attention, compromised executive function, and memory issues in older adults with T2DM [38].

The activity of the insulin-degrading enzyme (IDE) is impaired due to irregular signaling from insulin receptors, which affects downstream processes. IDE's main role is to break down insulin and amyloid-beta(Aβ) proteins, helping to regulate their levels effectively. However, in disease states, the IR competes with Aβ for IDE binding, leading to reduced IDE function. This reduction in Aβ degradation in the brain significantly contributes to the onset of AD. Hon et al. [39] demonstrated that glycogen synthase kinase-3β(GSK-3β) is involved in the hyperphosphorylation of tau protein. Lesort et al. [40] found that both GSK-3β and synthase kinase-3α (GSK-3α) are influenced by insulin in a dual regulatory manner, affecting their activity. The insulin/glucagon signaling pathway is vital for balancing the phosphorylation and dephosphorylation of tau proteins, which is crucial for normal cellular operations and various physiological functions [41]. Furthermore, serine/threonine protein kinase B, known as Akt/PKB, has been recognized as a key inhibitory kinase for GSK-3β at the Ser9 site. When the Akt/PKB pathway is activated, it phosphorylates GSK-3β, leading to a decrease in its enzymatic activity. This highlights the significance of the Akt/PKB signaling pathway in regulating the functions of essential proteins involved in cellular signaling [42]. At the same time, this pathway's activation boosts IDE activity, aiding in the breakdown and clearance of Aβ. Therefore, it is proposed that IDE may influence tau protein phosphorylation levels [43], providing a theoretical basis for a better understanding of the mechanisms underlying DCI.

### 3.3 Hyperglycaemia

In typical physiological circumstances, glucose is the primary energy source for the brain, especially in areas like the hippocampus, which is particularly sensitive to changes in glucose levels. While the exact ways in which high blood glucose levels may lead to dementia are still under investigation, it is likely that such levels heighten the risk of developing this condition. Studies indicate that experiencing diabetes in middle age can lead to cognitive deterioration as one grows older. Additionally, individuals with high HbA1c levels often show diminished cognitive abilities [44, 45]. Research has shown that prolonged high blood sugar can negatively impact both the structure and function of the brain. This ongoing state of hyperglycemia results in two major pathological consequences. First, hypoxic metabolism worsens acidosis, potentially harming the central nervous system (CNS) and neurons. Due to the high-sugar state, there are structural changes in the microcirculatory system, including decreased endothelial function, damage to endothelial cells, and thickening of the capillary basement membrane. These changes not only lead to a decrease in perfusion within the tissues and a reduction in blood and oxygen supply but also result in impaired oxygen diffusion, further exacerbating tissue hypoxia. Secondly, elevated blood sugar promotes the production of advanced glycation end products (AGEs) through pathways such as the polyol and hexosamine pathways. AGEs are a varied group of compounds that form when macromolecules like proteins, lipids, or nucleic acids react with reducing sugars [46]. Over time, as diabetes progresses and aging occurs, these compounds accumulate. When AGEs bind to cell surface receptors, they can cause cytotoxic effects. The interaction of AGEs with the receptor for advanced glycation end-products (RAGE) activates the diacylglycerol (DAG) protein kinase C (PKC) pathway. In conditions of high glucose, this binding event triggers the production of DAG or facilitates the breakdown of phosphatidylcholine, which activates PKC. This activation results in the generation of a significant amount of reactive oxygen species (ROS) within the cell, disrupting the balance between oxidation and antioxidation, ultimately leading to cell death [47]. Hyperglycemia disrupts brain energy metabolism, synaptic function, and BBB homeostasis through a multi-pathway interaction of "metabolic toxicity - vascular injury - neuroinflammation - OS," ultimately leading to CD.

### 3.4 Hypoglycaemia

Glucose serves as the main energy source for the human body, and the brain lacks the ability to generate or store it. Thus, maintaining a steady supply of glucose is crucial for cognitive health. Temporary drops in blood sugar can result in short-lived cognitive issues, while chronic or severe hypoglycemia may lead to permanent neuronal damage. Such damage can alter brain structure and adversely affect cognitive abilities [48]. Recent studies indicate that CD linked to low glucose levels may involve multiple mechanisms. One of these is the release of excitatory neurotransmitters, diminished glycolysis from low glucose can significantly increase the release of aspartate and glutamate. This process activates NMDA receptors, a specific type of ionotropic glutamate receptor, leading to the opening of sodium-calcium channels. As a result, ionic imbalances in neuronal cells can occur, causing mitochondrial dysfunction and ultimately resulting in neuronal death [49, 50]. Mitochondria are located along axons, in presynaptic terminals, and within dendrites of neurons. The disruption of mitochondrial function, along with synaptic damage and CD, is recognized as a pathological characteristic of brain injury in individuals with T2DM [51]. Lin et al. [52] showed that hypoglycemic diabetic mice experiencing repeated low blood sugar episodes, combined with chronic high blood sugar, increased the production of ROS, disrupted mitochondrial membrane potential, and impaired mitochondrial energy production, which significantly affected the mice's long-term recognition and spatial memory. However, restoring mitochondrial structure and function improved cognitive performance in these mice. These findings suggest that OS in mitochondria and synaptic damage due to low blood sugar may significantly contribute to cognitive dysfunction.

### 3.5 Vascular lesions

Vascular lesions are a severe form of CD associated with cerebrovascular issues. A major risk factor for VaD is DM [53]. Individuals with T2DM are at an increased risk for various vascular complications, such as cerebral arteriolosclerosis, ischemic white matter loss, large vessel atherosclerosis, lacunar infarctions, thromboembolic events, hemorrhagic strokes, and subarachnoid hemorrhages, compared to those without diabetes. These vascular conditions elevate the chances of developing VaD. The coexistence of both microvascular and macrovascular complications in diabetic patients plays a significant role in cognitive deterioration. Diabetics often experience both localized and systemic vascular issues. Research indicates that DM can double to quadruple the risk of coronary artery disease (CAD), peripheral arterial disease (PAD), and cerebrovascular disease [54]. The vascular complications seen in diabetic individuals are associated with alterations in metabolism and blood circulation [55]. Studies reveal that diabetic patients with cerebrovascular disease have a twofold higher risk of CD than those without such conditions (hazard ratio (HR) 2.03; 95% confidence interval (CI) 1.88-2.19) [56]. Diabetic retinopathy (DR) is a marker of microvascular damage in the brain and is associated with CD [57]. Furthermore, research shows that individuals with diabetes and retinopathy are nearly three times more likely to suffer from CI than those without retinopathy, highlighting the significant impact of microvascular brain damage on cognitive function.

### 3.6 Lipid metabolism dysfunction

**Figure 2.**
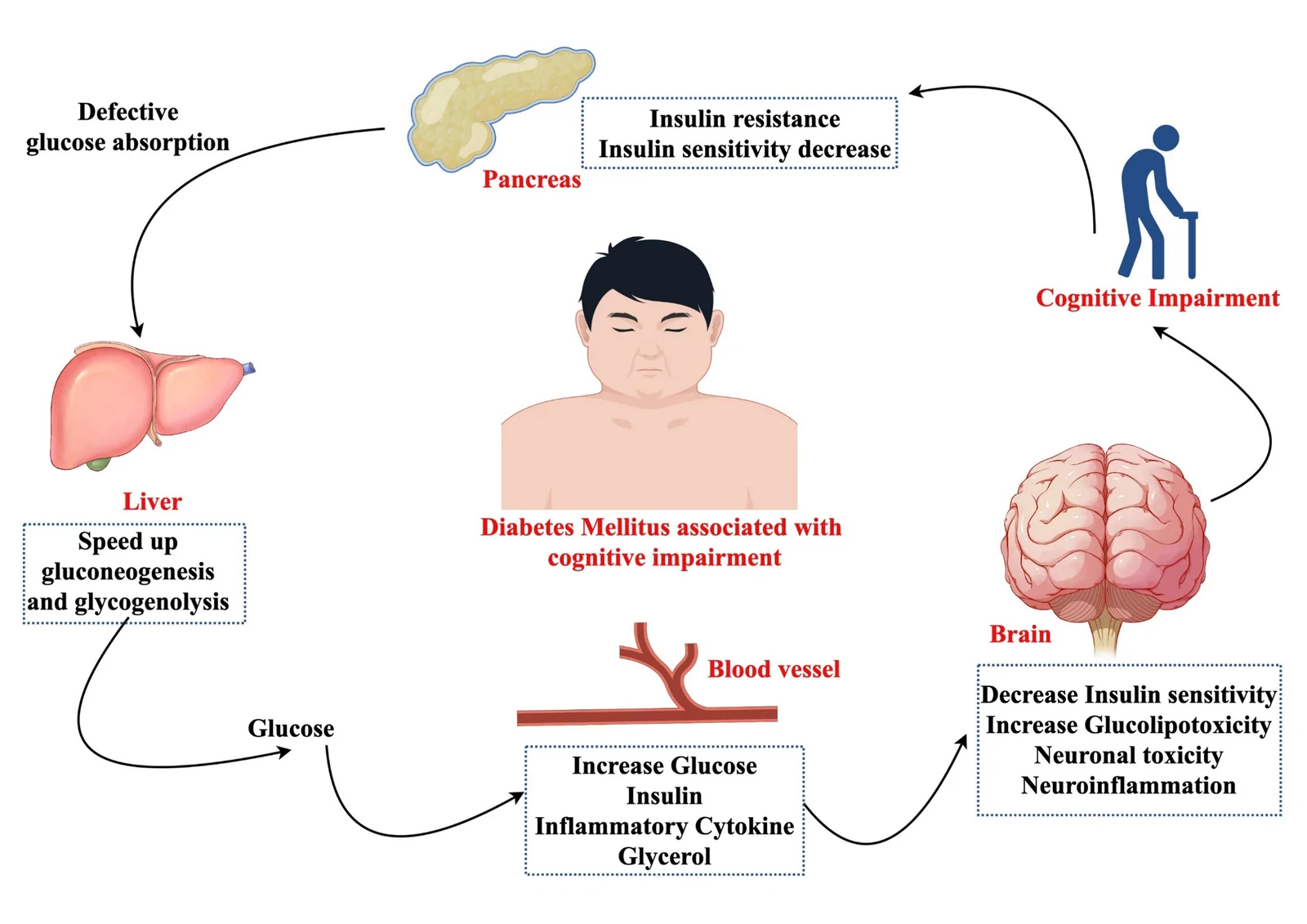
**Impaired blood glucose metabolism in T2DM-induced dementia**. Patients with dementia from T2DM exhibit systemic hyperglycemia, hyperlipidemia, and hyperinsulinemia. Decreases in IR and insulin sensitivity are characteristics of T2DM. A lack of insulin sensitivity prevents the liver from absorbing and using glucose from the blood. Defective glucose absorption encourages the liver to speed up gluconeogenesis and glycogenolysis, which raises the level of glucose in the blood. Finally, the brain also displays IR with excess glucose through blood, which contributes to increasing neuronal damage and neuroinflammation.

Individuals with T2DM often exhibit signs that are associated with disruptions in lipid metabolism. These metabolic irregularities play a significant role in the complications related to DCI (Fig. 2). Studies have revealed a relationship between apolipoprotein B, low-density lipoprotein (LDL), and cognitive deterioration [58]. Furthermore, reduced levels of high-density lipoprotein (HDL) are associated with elevated Aβ levels in the brain [59]. Moreover, administering medium-chain triglycerides orally has demonstrated beneficial effects on cognitive abilities in AD patients [60].

Apolipoprotein E (APOE), produced by astrocytes in the brain, is essential for the incorporation of cholesterol into neurons and aids in converting amyloid precursor protein into Aβ [61]. On a broader scale, high cholesterol levels may contribute to Tau accumulation in the brain [62], potentially leading to or worsening CD. Additionally, APOE serves as a vital antioxidant. Neurobiological studies indicate that the ApoE protein is significantly involved in the advancement of AD [63]. The *ApoE* gene, located on chromosome 19, is the main genetic risk factor linked to sporadic AD and has three variants: *ApoEε2*, *ApoEε3*, and *ApoEε4* [64]. Research shows that individuals with the *ApoEε4* allele have a heightened risk of developing AD, often experiencing an earlier onset, which highlights its role as a genetic vulnerability [65, 66]. Notably, over 40% of those with sporadic Alzheimer's possess the *ApoEε4* variants [67]. Having one *ApoEε4* allele (heterozygous) increases the risk of sporadic AD by about 3.7 times compared to those with the *ApoEε3* allele, indicating that even a single copy of *ApoEε4* can significantly affect an individual's risk for this neurodegenerative disorder. Moreover, individuals with two *ApoEε4* alleles (homozygous) face an even greater risk, with studies suggesting their chances of developing sporadic AD are approximately 12 times higher. This significant disparity highlights the influence of genetic factors on the likelihood of AD, underscoring the importance of genetic testing and risk evaluation in assessing an individual's susceptibility to this condition [68,69].

The *APOE* gene also mediates CI in diabetic patients. The binding of APOE to its receptors activates dual leucine zipper kinase (DLK), leading to the phosphorylation of extracellular signal-regulated kinase 1/2 (ERK1/2). The activated ERK1/2 further induces the phosphorylation of the transcription factor c-fos, stimulating the activation of the transcription factor activator protein-1 (AP-1), which enhances the transcription of β-APP, thereby increasing the deposition of Aβ. Research has shown a link between the *ApoE ε4* allele and Aβ within amyloid plaques, indicating a relationship between these elements and AD. The *ApoEε4* variant significantly raises the risk of developing AD, even when accounting for factors like age, smoking, alcohol use, and cardiovascular health [70]. Mechanistically, the *ApoEε4* allele is less proficient at degrading Aβ peptides compared to other variants, which contributes to a greater chance of Aβ plaque formation [63, 71]. This suggests a connection between overall lipid levels and DCI, although the specific roles of different lipid types in CI vary.

### 3.7 Microbiota-Gut-Brain axis (MGBA) dysfunction

**Figure 3.**
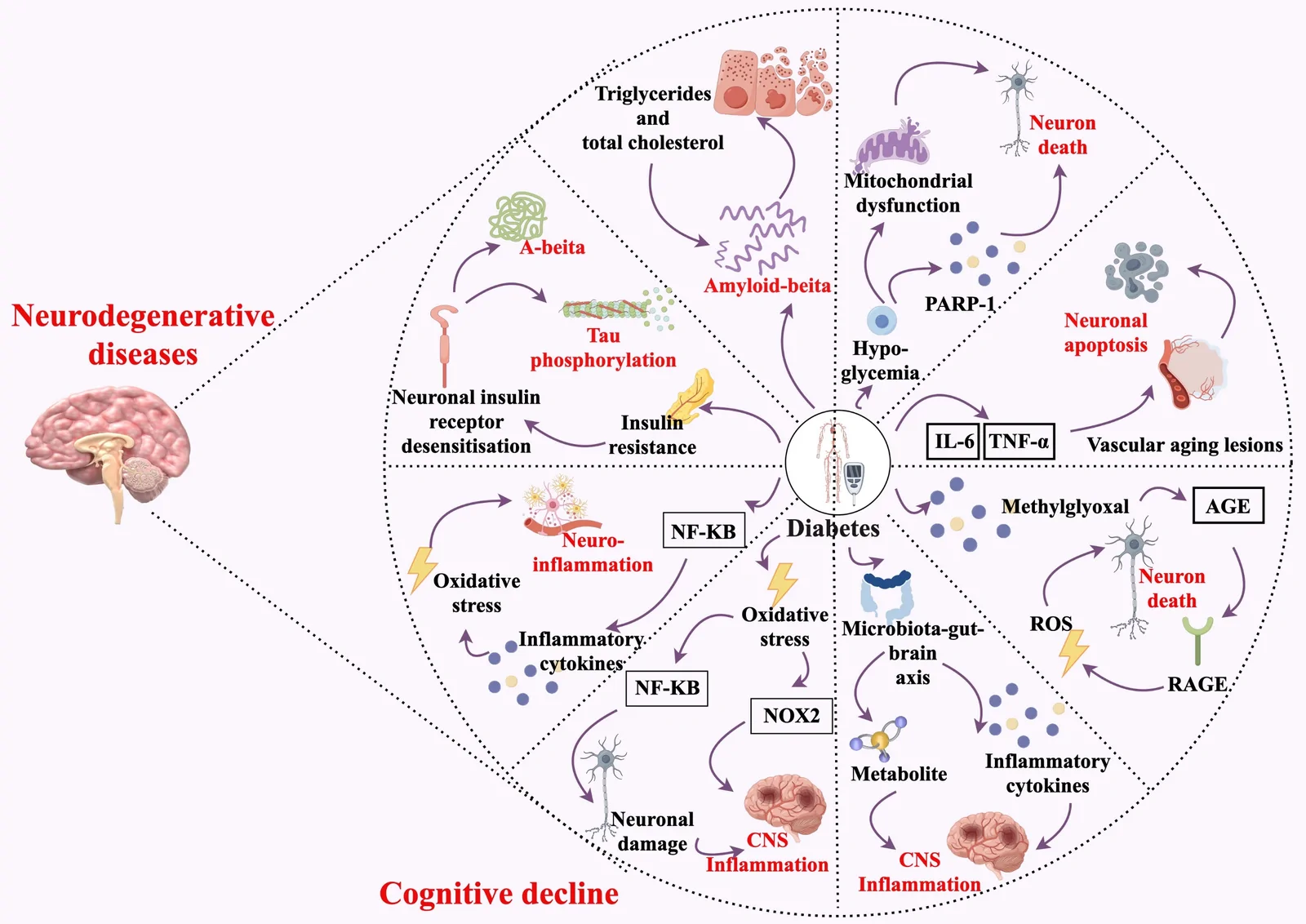
DM is associated with cognitive impairment.

The host's gut microbiome primarily affects it through the bacteria present and their metabolic byproducts (Fig. 3). Studies indicate that altering diet to boost beneficial gut bacteria can enhance cognitive function in mice [72]. When bacterial metabolism is disrupted, it can lead to endotoxemia, which causes inflammation, diminishes the intestinal barrier, and increases the BBB's permeability, adversely impacting cognitive abilities. Furthermore, the gut microbiota can directly promote the release of pro-inflammatory cytokines [73]. Krabbe et al. [74] showed that administering lipopolysaccharide (LPS) intravenously to healthy individuals resulted in elevated serum inflammatory cytokines and decreased memory performance. The ability of these cytokines to access the brain highlights their crucial role in gut-brain signaling [75]. Regarding the intestinal barrier's integrity, both LPS and pro-inflammatory cytokines have been found to weaken it [76, 77], while antibiotics may improve the intestinal barrier's function and safeguard the BBB's integrity [78].

Research has shown that people with DCI exhibit lower levels of glucagon-like peptide-1 (GLP-1) and peptide YY (PYY) [79].This decrease seems to be significantly affected by the gut microbiome and its metabolites, indicating that these factors may play a role in DCI. Additionally, changes in the gut microbiome could affect the production of neurotransmitters and neuropeptides vital for cognitive abilities, such as brain-derived neurotrophic factor (BDNF) and gamma-aminobutyric acid (GABA). Evidence suggests that Bifidobacterium spp. can boost BDNF gene expression in the hippocampus of mice, while those with DCI show a notable reduction in these beneficial bacteria [80]. This drop in BDNF levels may be a critical factor in the onset of DCI. Moreover, the gut microbiome might also affect GABA levels, which are crucial for learning memory [81]. Overproduction of glutamate can lead to increased neuronal activity, resulting in neurotoxicity and CD. GABA acts as an inhibitory neurotransmitter, modulating glutamate activity and contributing to neuroplasticity signaling pathways. Studies indicate that the functioning of GABAergic neurons can affect synapse formation and strengthening, thereby facilitating long-term potentiation (LTP), a fundamental process for learning and memory.

Short-chain fatty acids (SCFAs) like propionate, sodium butyrate, and acetate are byproducts formed when gut bacteria ferment dietary fiber [82]. These crucial substances are involved in various physiological functions, such as glycolipid metabolism, immune system regulation, and the gut-brain axis's operation [83, 84]. Li et al. [72]indicated that altering diets to boost beneficial gut bacteria and their SCFA production can improve glycolipid metabolic processes in mice. This improvement helps protect hippocampal neurons from damage, thereby enhancing cognitive abilities. Acetate, in particular, is a key energy source and a metabolic marker for astrocytes. It can cross the BBB and affect appetite control by activating central homeostatic mechanisms. Studies have shown that lower acetate levels are linked to decreased synaptophysin in the hippocampus, a protein crucial for synaptic plasticity and learning [85]. Notably, Zheng et al. [86]discovered that a long-term lack of acetic acid in diabetic mice resulted in lower synaptophysin levels in the hippocampus, leading to CD. However, external administration of acetic acid was found to alleviate these cognitive issues. Additionally, reduced propionate levels in the blood can affect liver metabolism, particularly in glucose and lipid management. This SCFA promotes glycolysis and mitochondrial respiration in astrocytes [86, 87], which may trigger neuroinflammation and facilitate the conversion of astrocytes into the neuroprotective A2 subtype, enhancing their mitochondrial function [88]. Furthermore, trimethylamine N-oxide, a compound linked to gut-brain interactions, can induce pro-inflammatory responses in astrocytes, diminishing their metabolic adaptability, disrupting normal brain energy metabolism, and weakening the BBB, all of which increase the likelihood of CD [89].

### 3.8 Hypothalamic-pituitary-adrenal (HPA) axis

Studies have shown a notable link between an overly active HPA axis and cognitive deterioration in individuals with DM, especially in those with T2DM. This condition is marked by heightened HPA axis activity, leading to increased cortisol levels in the blood. Such hormonal disruptions are thought to interfere with synaptic plasticity in the hippocampus, a vital area of the brain responsible for memory and learning, potentially triggering neurodegenerative changes [90]. In a pivotal study, Sato et al. [91]uncovered that high cortisol levels can stimulate the formation of ROS, which are detrimental molecules that can cause oxidative harm to the hippocampus, ultimately resulting in neuronal cell death—a crucial aspect of neurodegeneration. Moreover, elevated glucocorticoid levels have been associated with the buildup of excitatory amino acids, particularly glutamate, in the hippocampus. This accumulation leads to persistent activation of NMDA receptors, worsening the energy shortfall in neurons. Additionally, Bruehl et al. [92] involving 30 T2DM patients found evidence of HPA axis dysfunction related to memory issues. Their results indicated a negative correlation between cortisol levels and memory performance, suggesting that higher cortisol may be linked to diminished cognitive abilities. Other investigations have also pointed out that increased glucocorticoids can heighten the neurotoxicity of Aβ and facilitate tau protein phosphorylation, which can trigger neuronal apoptosis. Dey et al. [93]further explored this in a db/db mouse model, discovering that elevated cortisol in the hippocampus activated GSK-3β, resulting in excessive tau phosphorylation and subsequent learning and memory deficits [94]. These findings collectively highlight a possible association between HPA axis dysfunction and CD in diabetic individuals, although further studies are needed to clarify this intricate relationship.

### 3.9 Neurotrophic factors

Neurotrophic factors (NTFs), including BDNF and nerve growth factor (NGF), are essential for the development and differentiation of cholinergic neurons, playing a crucial role in preventing neuronal degeneration. BDNF, in particular, significantly impacts various synaptic activities, neurotransmission, neuronal repair, and the overall adaptability of the CNS. It is primarily found in areas like the hippocampus and cortex, where it interacts with specific receptors to activate the tyrosine kinase B receptor (TrkB). This activation triggers the phosphorylation of TrkB, enhancing its biological functions and facilitating numerous neuronal processes [95]. The influence of NTFs goes beyond mere receptor interaction; these factors are integral to the growth and upkeep of neurons. They are absorbed by axon terminals and transported retrogradely via axoplasmic flow to the neuronal cytoplasm. This transport is crucial for fostering neuronal growth during development and maintaining the functionality and structural integrity of adult nerves, especially in the context of regeneration after injury. In diabetic patients, there is often a significant reduction in NGF and BDNF levels in the brain. This decrease can lead to an exaggerated response of NTFs to injury. However, such a weakened response hampers neurons' ability to combat challenges like ischemia, OS, and other harmful factors that contribute to cell death. As a result, this impaired response may cause considerable degenerative alterations in neuronal structure and function over time.

Endogenous BDNF plays a vital role in sustaining LTP, which is a key process in cognitive functions such as learning and memory. Research indicates that individuals with DCI show a more pronounced decrease in circulating BDNF levels compared to those with T2DM or AD alone. This suggests a possible connection between BDNF and the cognitive challenges faced by DCI patients [96]. Additionally, studies reveal a significant relationship between BDNF levels and both memory deficits and IR in T2DM patients [97]. The presence of high serum BDNF alongside diminished cognitive abilities in diabetics implies that BDNF could be an important biomarker for evaluating DCI's severity [98]. Furthermore, investigations have demonstrated that inhibiting the BDNF gene in mice results in considerable changes in the growth and function of striatal neurons. Together, these results highlight BDNF's critical involvement in the development of cognitive dysfunction in DCI patients, emphasizing its importance for neuronal well-being and cognitive function [99].

NGF is a crucial component of the neurotrophic factor family, playing a significant role in the growth, development, survival, and functionality of certain neurons in the CNS, such as cholinergic, sympathetic, and sensory neurons. A persistent lack of NGF negatively affects the operation of cholinergic neurons, which are vital for numerous cognitive processes [100]. Luo et al. [101]indicated that enhancing NGF levels could effectively alleviate cognitive dysfunction in rats modeled after AD, underscoring NGF's potential as a target for treating neurodegenerative disorders. Additionally, their study revealed that diabetic rats, induced with streptozotocin, had markedly reduced NGF levels in their brains compared to healthy controls, suggesting a link between NGF and diabetes-related nerve damage. In another study, mice genetically modified to be NGF-deficient developed senile plaques and neurofibrillary tangles, both of which are hallmarks of AD. These findings suggest that low NGF levels may not only hasten brain aging but also harm cholinergic neurons. Moreover, this NGF deficiency could interfere with LTP in the hippocampus, a critical mechanism associated with learning and memory.

### 3.10 Neuroinflammation

Neuroinflammation is a key factor in the progression of disorders affecting CNS, including AD and VaD [102]. This inflammatory reaction involves the activation of immune cells within the brain and the migration of immune cells from outside the CNS. Consequently, a range of pro-inflammatory cytokines, chemokines, and ROS are generated. This immune response compromises the BBB's integrity, leading to neuronal damage characterized by swelling, degeneration, and tissue death. Such pathological alterations significantly impair cognitive functions, contributing to the CD seen in these conditions [103-105]. Microglia, the brain's resident immune cells [106, 107], shift from a resting state to an active state in response to various stimuli. In their activated form, microglia can perform phagocytosis, allowing them to clear away excess apoptotic neurons and aid in the repair and reconstruction of neuronal networks [108, 109]. These cells extend their processes to engage with different elements of the neural structure, such as dendritic spines, axons, and synapses, thereby improving communication and connectivity in the brain [110]. Additionally, when microglial activity is reduced, there is a marked increase in the number of nearby dendritic spines. This observation highlights the crucial role of microglia in the structural refinement and development of neural components, emphasizing their significance in preserving cognitive health and fostering neural plasticity [111, 112].

Glutamate is a crucial excitatory neurotransmitter in the CNS, and recent studies have revealed a significant decline in the NMDA, which is closely linked to glutamatergic synaptic activity. This decrease in NMDA receptor presence indicates a possible change in synaptic functionality that may have extensive effects on neuronal communication. Electrophysiological research has shown that mice devoid of microglia, essential elements of the brain's immune defense, display reduced frequencies of NMDA receptor-mediated excitatory postsynaptic currents. This observation highlights the critical role of microglia in sustaining normal excitatory synaptic transmission. Astrocytes, the predominant type of glial cells in the brain, not only offer vital support and insulation to neurons but also play a key role in forming and upholding the BBB [113]. Studies have pointed out that neuroinflammation involving astrocytes is a significant contributor to the buildup of amyloid plaques, neuronal degeneration, and synaptic dysfunction linked to AD [114, 115]. In the context of chronic neuroinflammation seen in the brains of AD patients, astrocytes display a dual role, exhibiting both protective and harmful traits. These opposing functions are significantly shaped by the release of specific activating factors during the early stages of inflammatory responses [116, 117]. Typically, astrocytes help safeguard neuronal cells by boosting the production of glutathione, a vital antioxidant. However, inflammatory processes can inflict oxidative damage on astrocytes, leading to an increase in ROS in AD, which may undermine their protective function and further promote neurodegeneration [118].

Research has shown that individuals with diabetes often experience a persistent systemic inflammatory condition, marked by heightened levels of inflammatory indicators such as ultrasensitive C-reactive protein, interleukin-6 (IL-6), and tumor necrosis factor-alpha (TNF-α) [119, 120]. A cross-sectional analysis revealed a significant correlation between elevated plasma concentrations of IL-6 and TNF-α and diminished cognitive abilities [121]. Esmaeili et al. [122]noted that T2DM and sporadic AD negatively impact memory and increase TNF-α and IL-6 levels in the hippocampus of rats. The stimulator of interferon genes (STING) signaling pathway has been confirmed as a core component of the intrinsic immune system in animal cells, capable of being activated in the event of DNA damage. The cyclic GMP-AMP synthase (cGAS)/STING pathway can accelerate the aging process through various mechanisms. In senescent cells, the expression of three prime repair exonuclease 1 (TREX1) and deoxyribonuclease 2 (DNase2) is downregulated, leading to the accumulation of DNA in the cytoplasm, which activates the cGAS/STING pathway, promotes the secretion of IFN-β, and subsequently drives the formation of the secretory-associated senescence phenotype (SASP). In aging microglia, mitochondrial homeostasis is disrupted, and mtDNA leakage activates the cGAS/STING pathway, triggering neurodegeneration and CD. Pretti et al. [123] found that C-176, a specific STING inhibitor, reduces levels of cGAS, p-STING, IFN-β, and pro-inflammatory markers linked to microglia/M1, such as cluster of differentiation 16 (CD16), C-X-C motif chemokine ligand 10 (CXCL10), TNF-α, and IL-1β mRNA in the brains of diabetic individuals. Thus, diminishing STING signaling may help protect against DCI. Increased levels of pro-inflammatory cytokines like TNF, IL-1, IL-2, and IL-6 have been detected in the brains of DCI patients [124], underscoring the role of inflammation in neuronal injury [125]. Additionally, factors such as OS, hyperinsulinemia, and hyperglycemia play a significant role in activating nuclear factor kappa B (NF-κB), which influences apoptosis, ROS production, and the regulation of TNF and IL expression, all crucial for inflammatory processes in neural cells [126]. Furthermore, inflammation has been linked to the disruption of the BBB, which may expose neurons to harmful substances and disrupt normal neural functions [127].

### 3.11 OS

OS is increasingly acknowledged as a significant factor in the development and advancement of DM and its related complications. A particularly alarming consequence of DM is DCI, which is heavily affected by oxidative damage mechanisms. Heightened OS can trigger lipid peroxidation, adversely impacting mitochondrial DNA. This damage interferes with mitochondrial function, essential for energy generation and neuronal well-being. As a result, mitochondrial dysfunction may lead to neuronal death, worsening DCI effects [128]. Caspersen et al. [129]revealed that mice with DCI exhibited marked reductions in mitochondrial enzyme activity in their brain cells. This decline is crucial as it leads to the buildup of oxidative agents, such as free radicals, capable of oxidizing cellular lipids, resulting in harmful aldehyde compounds. Several common biomarkers, including ROS, reactive nitrogen species (RNS), and peroxides, indicate the presence of OS. Additionally, byproducts of lipid peroxidation like malondialdehyde (MDA) and 4-hydroxynonenal (4-HNE), along with the DNA oxidation marker 8-hydroxy-2'-deoxyguanosine (8-OHdG), are vital for assessing oxidative damage levels. Ongoing research highlights the crucial role of OS in the pathways leading to DCI. Moreover, IR significantly influences DCI progression through various harmful effects. IR results in diminished glucose metabolism, increased AGEs, and the buildup of Aβ peptides and lipid deposits. These alterations contribute to mitochondrial dysfunction and disrupt energy metabolism, leading to an excess of ROS and RNS [130]. The brain's susceptibility to OS is heightened due to its high oxygen demand, abundant lipid content, and relatively low antioxidant enzyme levels. This combination makes the brain particularly vulnerable to oxidative damage, which can cause neuronal injury and the loss of synaptic connections, further aggravating CD in individuals with diabetes.

Studies have shown that high levels of blood sugar can greatly increase the generation of oxygen free radicals. This rise is attributed to the dysfunction of vital antioxidant enzymes, including superoxide dismutase and glutathione peroxidase, which are essential for neutralizing harmful reactive species. As a result, the reduced capacity of antioxidants can lead to neuronal cell death and damage, posing significant threats to neural health [131]. Additionally, the OS that ensues has been found to worsen inflammatory responses in the nervous system. This occurs through the activation of microglia and astrocytes, which are glial cells that respond to inflammation by releasing pro-inflammatory cytokines, thereby perpetuating a cycle of neuroinflammation. Specifically, ROS can disrupt critical signaling pathways such as NF-κB, c-Jun N-terminal Kinase (JNK), and Janus Kinase (JAK)/Signal Transducer and Activator of Transcription (STAT), leading to increased levels of inflammatory mediators and amplifying the overall inflammatory response [132]. Xu et al. [133]highlighted the impact of a high-fat diet, which was shown to cause significant IR, activate microglial cells, and stimulate the growth of astrocytes in a rat model. The study also noted a rise in OS alongside these changes. The results emphasized that chronic diabetes is linked to an intensified inflammatory response, marked by the over-activation of cytokines like TNF-α, IL-6, and IL-1β. This inflammatory cascade triggers JNK, which in turn leads to abnormal activation of Inhibitor of Nuclear Factor-κB (IKK) and Protein Kinase R (PKR). These cascading effects worsen IR by promoting the serine phosphorylation of Insulin Receptor Substrate-1 (IRS-1), crucial for maintaining proper hippocampal function. Furthermore, an imbalance between oxidative and antioxidant systems can further enhance the serine phosphorylation of IRS-1, disrupting the insulin signaling pathway. This disruption increases the expression of β-secretase genes, subsequently raising the production of Aβ, a peptide associated with CD [134]. Thus, it is clear that IR, inflammation, and OS related to T2DM are closely interconnected, with each factor affecting the others. This interaction not only leads to neuronal dysfunction but also plays a significant role in CD, highlighting the necessity of understanding these relationships in the context of diabetes and neurodegeneration.

### 3.12 Autophagy

Autophagy is an essential process for cellular degradation, involving the lysosomal breakdown of cytoplasmic elements. This mechanism plays a crucial role in protecting cells from various stressors such as oxidative damage, lack of nutrients, and harmful environmental factors. By engaging in autophagy, cells can effectively eliminate damaged or malfunctioning components, thus preserving homeostasis and overall cellular health [135]. There are several types of autophagy, including macroautophagy, microautophagy, and chaperone-mediated autophagy, each characterized by distinct features and roles. Typically, the term 'autophagy' is primarily associated with macroautophagy, which is characterized by the creation of double-membraned vesicles called autophagosomes. These vesicles are vital as they encapsulate damaged organelles and various molecules, transporting them to lysosomes. When autophagosomes merge with lysosomes, they create autophagolytic lysosomes, specialized compartments that facilitate the hydrolysis and breakdown of the enclosed materials. This process is crucial for recycling cellular components and maintaining proper cellular function [136].

#### 3.12.1 Microglia autophagy

In reaction to pathogen attacks, microglia take on a protective function by engulfing these invaders through autophagy and then delivering them to lysosomes for breakdown [137].Throughout the development and maturation of the CNS, microglia play a crucial role in managing synaptic formation, interaction, and adaptability through autophagic processes [138]. Additionally, autophagy is vital for facilitating the shift of microglia from a pro-inflammatory to an anti-inflammatory state, which aids in reducing neuroinflammation. Factors related to diabetes, such as high blood sugar and IR, can interfere with the autophagic functions of microglia. Autophagic dysfunction may occur due to improper initiation of the process or the inability of autophagosomes to fuse with lysosomes. When autophagy in microglia is compromised, their ability to clear neuroinflammatory agents, Aβ, and other harmful substances is diminished, leading to increased neuroinflammation and the buildup of toxic materials. This chain reaction adversely affects synaptic performance and neuronal communication, ultimately contributing to CD linked to diabetes.

#### 3.12.2 Mitochondrial autophagy

Mitochondrial autophagy represents a specialized type of autophagy crucial for the progression of DCI. This mechanism entails the identification and tagging of impaired mitochondria, which triggers the transformation of microtubule-associated protein 1 light chain 3 (LC3) into its membrane-bound form, referred to as LC3II. The presence of LC3II is critical for creating the double-membrane structure found in mature autophagosomes. It is then converted into the membrane-associated LC3III via the pre-autophagosome structure (PAS). During this sequence, LC3II interacts with mitochondrial marker proteins and phosphorylated ubiquitin, resulting in the formation of mitochondrial autophagosomes that ultimately merge with lysosomes for degradation [139]. In normal physiological conditions, mitochondrial autophagy plays a key role in maintaining cellular balance and ensuring mitochondrial functionality by selectively eliminating damaged mitochondria [140]. In contrast, under pathological conditions, a lack of mitochondrial autophagy can lead to an abnormal buildup of mitochondria and increased peroxide levels, potentially causing cellular damage [141]. Additionally, excessive mitochondrial autophagy may result in mitochondrial fragmentation and the release of lysosomal contents, which can ultimately lead to cell death [142].

A recent study examined the link between DM and MCI through proteomic analysis, revealing significant changes in proteins involved in the disrupted mitochondrial autophagy pathway. Key proteins such as optineurin (OPTN), sequestosome 1 (SQSTM1), and TBC1 domain family member 15 (TBC1D15) were highlighted. The findings indicated a crucial relationship: higher levels of OPTN, which plays a vital role in initiating mitochondrial autophagy, correlated with lower Mini-Mental State Examination (MMSE) scores. This suggests that mitochondrial autophagy, influenced by OPTN, may be activated in those with CI [143]. Carvalho et al. [144] explored mitochondrial function, synaptic health, and autophagic activity in the cortical and hippocampal areas of mice with T2DM, comparing them to 3xTg-AD model mice. Their results showed changes in the expression of Autophagy Related Protein 7 (ATG7) and lysosomal associated membrane protein 1 (LAMP1) in the lysosomal outer membrane, indicating a suppression of the autophagy-lysosome system, which is essential for cellular balance, especially under metabolic stress. Su et al. [145] contributed further evidence by showing that a high-glucose environment stimulates the expression of critical proteins for mitochondrial autophagy, namely PTEN-induced kinase 1 (PINK1) and E3 ubiquitin-protein ligase Parkin (Parkin). However, they also discovered that these high-glucose conditions impaired autophagic flux in a PC12 neuronal cell model, leading to a significant decrease in PINK1 and Parkin levels. This highlights how elevated glucose can disrupt PINK1/Parkin-mediated mitochondrial autophagy in neurons, potentially leading to cognitive deficits in diabetic individuals. Torres-Esquivel et al. [146] found that severe hypoglycemia hindered autophagic flow in the cerebral cortex and hippocampus of rats, as indicated by increased levels of LC3II and p62 proteins. This disruption in autophagic processes may contribute to neurodegenerative changes associated with diabetes, underscoring the importance of maintaining balanced glucose levels for brain health. Additionally, another study noted that inadequate insulin signaling, particularly in cases of IR, could impair mitochondrial function, resulting in increased OS and neuroinflammation, both of which are associated with CD in T2DM patients. The culmination of these pathological processes leads to neurodegeneration, highlighting the intricate relationship between diabetes, cognitive function, and the underlying cellular mechanisms involved [147]. Furthermore, another investigation revealed that DM patients experiencing cerebral ischemic injury (ischemia/ reperfusion, I/R) showed reduced mitochondrial autophagy and heightened OS in the later stages of the condition, contributing to neuronal cell death [148]. It is crucial to understand that both insufficient and excessive mitochondrial autophagy can worsen neuronal damage or lead to cell death. Therefore, conducting more comprehensive mechanistic studies is essential to clarify the factors influencing the various phenotypes of mitochondrial autophagy related to CI.

### 3.13 Metal ion metabolism disorders

New research indicates that imbalances in metal ions within the brain, particularly calcium, iron, and copper, are significantly associated with cognitive deterioration linked to diabetes. This disruption has been identified as a key element linking diabetes to neurodegenerative disorders, largely due to its role in processes related to OS, mitochondrial dysfunction, and injury to the BBB.

#### 3.13.1 Ca^2+^

Calcium ions (Ca^2+^) act as crucial second messengers that modulate various cellular functions by transmitting signals and activating a range of proteins, phospholipids, and enzymes involved in the breakdown of nucleic acids. Maintaining calcium equilibrium is vital for several processes, including growth, differentiation, action potential characteristics, synaptic plasticity, and memory formation. Disruptions in calcium levels within the brain significantly contribute to the mechanisms associated with DCI. DM can interfere with calcium homeostasis in neurons, leading to cell death due to an overload of calcium. This overload results from increased calcium entry, phospholipase activation, inhibition of mitochondrial electron transport, and the production of ROS, all of which contribute to neuronal impairment [149]. Voltage-gated calcium channels (Cav) are crucial for the entry of Ca^2+^ into cells and play a key role in various physiological functions. The Cav1.2 channel, the main type of L-type Ca^2+^ channels (LTCCs), is found in several organs and tissues, including the brain, heart, and smooth muscle. These channels are vital for numerous neuronal and physiological activities, especially in learning and memory. Research conducted by Singhal et al. [150] indicated that diabetic mice exhibited increased mRNA and protein levels of CaV1.2, which correlated with a notable decline in cognitive abilities. Treatment with nimodipine, an L-type calcium channel blocker (LCCB), improved synaptic plasticity and corrected the abnormal CaV1.2 levels in these mice, thus alleviating CI.

Numerous laboratory studies suggest that the endoplasmic reticulum (ER) acts as an additional storehouse for Ca2+, which may be vital for synaptic plasticity. It plays a significant role in Ca^2+^ storage and can release these ions in response to intense synaptic activity through intracellular Ca^2+^ release. In diabetic patients, disturbances in calcium regulation can result in atypical Ca^2+^-dependent synaptic plasticity, mitochondrial impairment, neuroinflammation, aggregation of Aβ, and hyperphosphorylation of tau protein, all of which may lead to CD. An excessive influx of extracellular Ca^2+^ into the ER and mitochondria can result in accumulation that triggers mitochondrial dysfunction and increased levels of ROS, leading to OS [151, 152]. The activation of G-protein-coupled calcineurin receptors initiates the NOD-like receptor pyrin domain-containing 3 (NLRP3) inflammasome via the myo-inositol/Ca^2+^ signaling pathway [153], which facilitates the release of IL-1β and other inflammatory cytokines that exacerbate neuroinflammation. Additionally, IR intensifies neuronal damage by causing a persistent mild activation of NMDA receptors, ultimately resulting in an overload of intracellular calcium [154]. Furthermore, Aβ enhances its own aggregation in response to calcium influx through various channels, which increases acetylcholinesterase activity and disrupts the regulation and metabolism of acetylcholine [153, 155]. Moreover, the activation of enzymes like calmodulin, which are involved in tau phosphorylation, contributes to the development of neurofibrillary tangles [153].

#### 3.13.2 Fe^2+^

Iron is a prevalent metal ion found in the brain [156, 157], playing a critical role in the production of nucleic acids, proteins, and neurotransmitters, as well as aiding in the development of neuromyelin [158, 159]. In physiological terms, Fe^3+^ is mainly delivered to cells through its binding with transferrin (Tf), which interacts with the transferrin receptor 1 (TfR1). After this complex is internalized by cells, Fe^3+^ can be stored in ferritin or converted to Fe^2+^, which is then released into the cytoplasmic labile iron pool (LIP) [160]. The excess Fe^2+^ in the LIP engages with phospholipids that contain polyunsaturated fatty acids (PUFA-PL) through the Fenton reaction, resulting in the production of lipid peroxides capable of initiating cellular ferroptosis [161, 162]. Hepcidin, produced by glial cells [163], influences ferroportin (FPN), the sole known protein responsible for the export of iron ions from cells. FPN is vital for regulating iron balance in the body by controlling the absorption, recycling, and storage of iron ions. The binding of hepcidin to FPN results in the degradation of FPN, which in turn reduces iron uptake and recycling.

Recent research indicates a notable connection between iron regulation and metabolic functions associated with T2DM. Díaz-López et al. [164] discovered a significant link between heightened serum ferritin levels and an increased risk of developing T2DM. In environments rich in glucose, pancreatic β-cells and isolated islet tissues from db/db mice exhibited signs of iron accumulation, mitochondrial impairment, and reduced insulin production [165]. Additionally, in a diabetes model induced by streptozotocin, both serum and islet tissue ferritin concentrations were significantly higher, leading to considerable damage to pancreatic β-cells, with electron microscopy revealing characteristics suggestive of ferroptosis in the mitochondria. These impacts may be intensified by agents that promote ferroptosis and mitigated by those that inhibit it [166]. *In vitro* studies have shown that excessive Fe2+ exposure in pancreatic β-cells can trigger ferroptosis, which may be reversed by the use of ferroptosis inhibitors [167]. Furthermore, research has indicated that iron accumulation is also present in neurodegenerative diseases like AD [168], where excess iron contributes to CD in vital areas such as the hippocampus, caudate nucleus, and thalamus, leading to MCIin the putamen[161]. Additionally, iron buildup in T2DM may play a role in the development of IR. Studies suggest that elevated insulin levels in the brain enhance iron uptake through TfR1, resulting in an overload of neuronal iron [169, 170]. Guo et al. [171] noted that erythropoietin (EPO) could help mitigate cognitive deficits by reducing iron overload, modulating the expression of ferroptosis-related proteins, and preventing ferroptosis itself. There is growing interest in the role of ferroptosis in the mechanisms underlying CI in diabetes [172], with research from Wang et al. [173] demonstrating that Gemfibrozil, a PPARα agonist, can inhibit ferroptosis and reduce the associated buildup of lipid peroxides and ROS, thereby enhancing cognitive function in db/db mice.

#### 2.13.3 CU^2+^

Copper (Cu) serves as an essential cofactor for the brain's antioxidant enzymes, significantly influencing energy metabolism, antioxidant defense, and neurotransmitter synthesis [174]. Sufficient copper levels are vital for the proper development of the human nervous system [175]. Conversely, excessive copper can greatly enhance cellular autotoxicity, potentially resulting in cell death. Copper ion transporters facilitate the entry of Cu^2+^ into cells, where it interacts with ferric oxide reducing protein 1 (FDX1) within the mitochondria. FDX1 triggers the thioctanoylation of dihydrolipoic acid amide S-acetyltransferase (DLAT). Concurrently, Cu^2+^ is converted into the more harmful Cu+, leading to the formation of ROS via the Fenton reaction. The ROS generated include superoxide anion (O2-), hydrogen peroxide (H2O2), hydroxyl radical (OH-), and nitric oxide (NO) [176, 177]. Monovalent copper ions bind to thiooctylated DLAT proteins, promoting their aggregation and resulting in the degradation of iron-sulfur cluster proteins. This intensifies proteotoxic stress and ultimately leads to cell death [178]. An increase in copper consumption has been linked to a deterioration in overall language abilities [179]. Moreover, higher serum copper levels have been noted in older adults with CD [180]. Research on animals has shown that copper chelators can alleviate cognitive deficits and behavioral issues in the R6/2 mouse model of Huntington’s disease [181]. Meta-analyses reveal that individuals with T2DM and AD have elevated serum copper levels [182-184], indicating a potential role in the onset of DCI. The association between abnormal copper metabolism and DCI is closely related to OS. In diabetic patients, increased copper levels may expedite the formation and accumulation of AGEs, intensify OS and ROS production, and disrupt synaptic plasticity [185].

Recent research indicates that copper may play a role in interacting with key pathogenic factors such as Aβ and Tau, potentially worsening the progression of the disease [186]. Studies have shown that individuals with AD have markedly elevated levels of both total and free copper in their bloodstream compared to those without the condition. Additionally, increased copper levels have been found in age-related lesions associated with AD. Excess copper can bind to Aβ, facilitating its aggregation and the formation of Aβ oligomers, which heightens neurotoxicity [186, 187]. In experiments involving BV-2 cells, copper has been found to boost the release of inflammatory substances like NO and TNF-α, while simultaneously reducing neurotransmitter levels such as serotonin (5-HT), dopamine (DA), and GABA in the brain tissue of mice [188]. Moreover, excess copper hinders the body's natural processes for clearing Aβ [189]. Kitazawa et al. [190]demonstrated that the Cu-Aβ complex diminishes the expression of LDL receptor-associated protein 1, which in turn disrupts the clearance of harmful Aβ oligomers. At the same time, copper promotes the self-aggregation and hyperphosphorylation of Tau proteins, contributing to the development of neurofibrillary tangles [191]. The use of copper chelators to reduce copper levels in AD mouse models has shown encouraging outcomes in reducing Aβ buildup and improving cognitive function in these animals [192].

Recent studies on the interplay between copper levels and coproptosis in CNS disorders have attracted considerable interest. Understanding the underlying pathogenic mechanisms has opened up new possibilities for drug treatments in DCI associated with these disorders. Upcoming therapeutic strategies are expected to emphasize the use of copper ion transporters and chelation therapies to manage copper metabolism and reduce coproptosis as the disease advances. Careful oversight of these processes could lead to significant advancements in treatment methodologies.

## 4. Discussion

DM and CD are major chronic health issues affecting people globally. Those diagnosed with diabetes are at a significantly higher risk of CI, which can diminish essential self-management abilities. This decline in cognitive function increases reliance on caregivers, creating a challenging situation for those impacted. As cognitive skills deteriorate, managing diabetes becomes more difficult, worsening the disease's progression and establishing a detrimental cycle where each condition adversely affects the other. Research from the Rotterdam study has shown that individuals with T2DM are nearly twice as likely to develop dementia compared to non-diabetic individuals. This concerning statistic highlights the serious effects of diabetes on cognitive health and emphasizes the necessity for enhanced awareness and management approaches [193]. In 1966, Nielsen et al. [194]performed autopsies on 16 diabetic patients with cognitive issues, revealing significant degenerative changes in both grey and white matter, which led to the concept of 'diabetic encephalopathy.' Epidemiological studies have confirmed a strong link between DM and two specific dementia types: AD and VaD. This connection is influenced by various factors, including age and genetic predispositions, indicating that the risk of CD can vary widely among diabetic individuals. This body of research underscores the urgent need for healthcare professionals to monitor cognitive health in diabetes patients, enabling early intervention strategies to reduce the risks associated with CD. The 2021 guidelines from the ADA stress the importance of acknowledging the relationship between DM and CD, proposing that diabetes may fundamentally be viewed as a brain disorder. Furthermore, these guidelines suggest that inadequate glycemic control correlates with CI [195]. According to the World Alzheimer's Disease Report (2015), around 50 million people globally are affected by dementia, with a new case diagnosed every three seconds, and approximately 7% to 13% of these cases are linked to diabetes. Thus, understanding the mechanisms behind DCI is vital for enabling early detection and intervention, which could help delay CD. Currently, treatments for AD mainly focus on alleviating symptoms and offer limited therapeutic advantages [196], with a lack of targeted clinical trials for dementia therapies specifically for DCI patients. However, due to the gradual nature of DCI progression, the outlook for many individuals, especially those under 60, is generally positive. A comprehensive understanding of DCI's pathogenesis could lead to the creation of strategies that may slow its progression and postpone the onset of cognitive difficulties.

IR, inadequate insulin secretion, and variations in blood sugar levels—both high and low—are crucial elements that lead to the onset of DCI. Moreover, issues such as blood vessel complications, neuroinflammation, oxidative damage, lipid metabolism irregularities, neurotrophic factor imbalances, autophagy dysfunction, and disturbances in metal ion metabolism play significant roles in this condition. These elements do not function independently, rather, they interact to create a complex pathological network that underpins DCI. A comprehensive exploration of DCI's pathogenesis is essential for establishing a solid theoretical basis for its diagnosis, treatment, and prevention. Understanding these mechanisms not only aids in developing more accurate diagnostic tools for the early detection and management of DCI, thus slowing its progression, but also paves the way for innovative therapeutic strategies targeting various underlying issues. For example, by altering the insulin signaling pathway, improving glucose metabolism, reducing OS and neuroinflammation, and managing metal ion metabolism, we can expect more focused and effective treatment options for individuals affected by DCI.

Recent studies have increasingly focused on improving the brain's ability to eliminate metabolic waste, especially in patients with AD. Researchers have found that linking this process to the cervicocranial lymphatic system offers notable clinical benefits, including both immediate relief and long-term positive effects. These discoveries highlight the lymphatic system's potential role in alleviating Alzheimer's symptoms, paving the way for new therapeutic strategies aimed at enhancing brain health. Additionally, it has been shown that DM directly impacts the structural integrity of the glial lymphatic system. This condition modifies the spatial properties of the perivascular spaces (PVS), which are vital for the circulation of cerebral fluids around small arterioles, capillaries, and veins. Moreover, DM negatively affects astrocytes and disrupts the polarization of aquaporin-4 (AQP4), a key protein necessary for maintaining water balance in the brain. Research indicates that alterations in the spatial structure of the PVS are closely linked to inflammatory responses and the development of various neurological disorders, emphasizing the complex relationships between metabolic diseases and brain health [197]. Astrocytes are not only integral components of the glial lymphatic system but also play a crucial role in glucose uptake across the BBB. Under conditions of elevated glucose, astrocytes exhibit overexpression of complement C3 both *in vivo* and *in vitro* [198]. This heightened expression of C3 worsens synaptic loss, ultimately resulting in CD [199, 200]. AQP4, a water channel protein found abundantly in astrocytic end-feet, is crucial for lowering the resistance of cerebrospinal fluid (CSF) in the brain's mesenchyme and PVS [201]. In diabetic rat models, the polarity of hippocampal AQP4 is compromised, which has been linked to a reduced capacity for clearing metabolic waste from the brain [202, 203]. Research into the role of the glial lymphatic system concerning DCI is still evolving. Additionally, there is a significant gap in longitudinal studies that explore the pathophysiological changes associated with the condition. Furthermore, there is limited research comparing how the glial lymphatic system interacts with T1DM and T2DM [204]. Given the various complications associated with diabetes, such as increased blood pressure and elevated lipid levels that may also affect the glial lymphatic system, it is crucial to address these factors in future research initiatives.

Future research directions for DCI should focus on addressing several issues: such as identifying early biomarkers for DCI, determining optimal blood glucose control targets for cognitive protection, and implementing personalized assessments and interventions to reduce the incidence of DCI. Cross-sectional studies should be conducted to evaluate the current cognitive status of diabetic patients and associated factors, with the aim of controlling these factors to delay or prevent CD, thereby improving patients' quality of life. Additionally, cohort studies should be undertaken to track the dynamic changes in patients' conditions. Interventional studies, such as randomized controlled trials, could also be conducted to test the efficacy of interventions like intensified blood glucose control and cognitive training. Furthermore, promoting interdisciplinary collaboration is essential, such as fostering the integration of endocrinology, neurology, geriatric medicine, and other related fields, to provide more comprehensive and precise prevention and treatment strategies for DCI.

To conclude, DCI is a significant complication associated with DM. A thorough understanding of its fundamental mechanisms can facilitate early detection and treatment, potentially delaying the onset of CD. Consequently, strong clinical trials will be essential in the future to enhance preventive measures and treatment strategies for those affected by DCI.

## Data Availability

All of the data used to support the findings of this study are included within the article.
